# Clinical use of lactate monitoring in critically ill patients

**DOI:** 10.1186/2110-5820-3-12

**Published:** 2013-05-10

**Authors:** Jan Bakker, Maarten WN Nijsten, Tim C Jansen

**Affiliations:** 1 Department of Intensive Care Adults, Erasmus MC University Medical Center, PO Box 2040, Room H625, Rotterdam, CA 3000, Netherlands; 2 Department of Critical Care, University of Groningen, University Medical Center, Groningen, Netherlands

## Abstract

Increased blood lactate levels (hyperlactataemia) are common in critically ill patients. Although frequently used to diagnose inadequate tissue oxygenation, other processes not related to tissue oxygenation may increase lactate levels. Especially in critically ill patients, increased glycolysis may be an important cause of hyperlactataemia. Nevertheless, the presence of increased lactate levels has important implications for the morbidity and mortality of the hyperlactataemic patients. Although the term lactic acidosis is frequently used, a significant relationship between lactate and pH only exists at higher lactate levels. The term lactate associated acidosis is therefore more appropriate. Two recent studies have underscored the importance of monitoring lactate levels and adjust treatment to the change in lactate levels in early resuscitation. As lactate levels can be measured rapidly at the bedside from various sources, structured lactate measurements should be incorporated in resuscitation protocols.

## Review

### Introduction

Many variables measured in critically ill patients have been used to estimate severity of disease, prognosticate morbidity and mortality, evaluate costs of treatment, and finally indicate specific treatment and monitor the adequacy of treatment and its timing. It is unlikely that one measurement can replace all of these, but in the remainder of this manuscript we will show that lactate levels may come close. Although in our mind strongly linked to tissue hypoxia, lactate levels follow many more metabolic processes not related to tissue hypoxia and, therefore, subject to many disturbances found in various clinical situations.

### History of lactate

The first description of lactate originates from 1780 when Karl Scheele found lactate in sour milk. It took almost 70 years before the German physician-chemist Joseph Scherer demonstrated the presence of lactate in human blood. Where Scherer analysed blood drawn from a young woman who had just died from what we now call septic shock, it was Carl Folwarczny in 1858 who demonstrated the presence of lactate in the blood of a living patient [1]. Araki and Zillessen made an important observation that has shaped our association of increased lactate levels and tissue hypoxia. These authors observed that when they interrupted oxygen supply to muscles in mammals and birds, lactic acid was formed and increased [2]. In current practice, lactate is frequently measured in many kinds of patients, usually with the goal of detecting tissue hypoxia. However, given the metabolism of lactate and the effect of acute illness on glucose metabolism, increased lactate levels can reflect more than only tissue hypoxia.

### Metabolism of lactate

**Figure 1:**
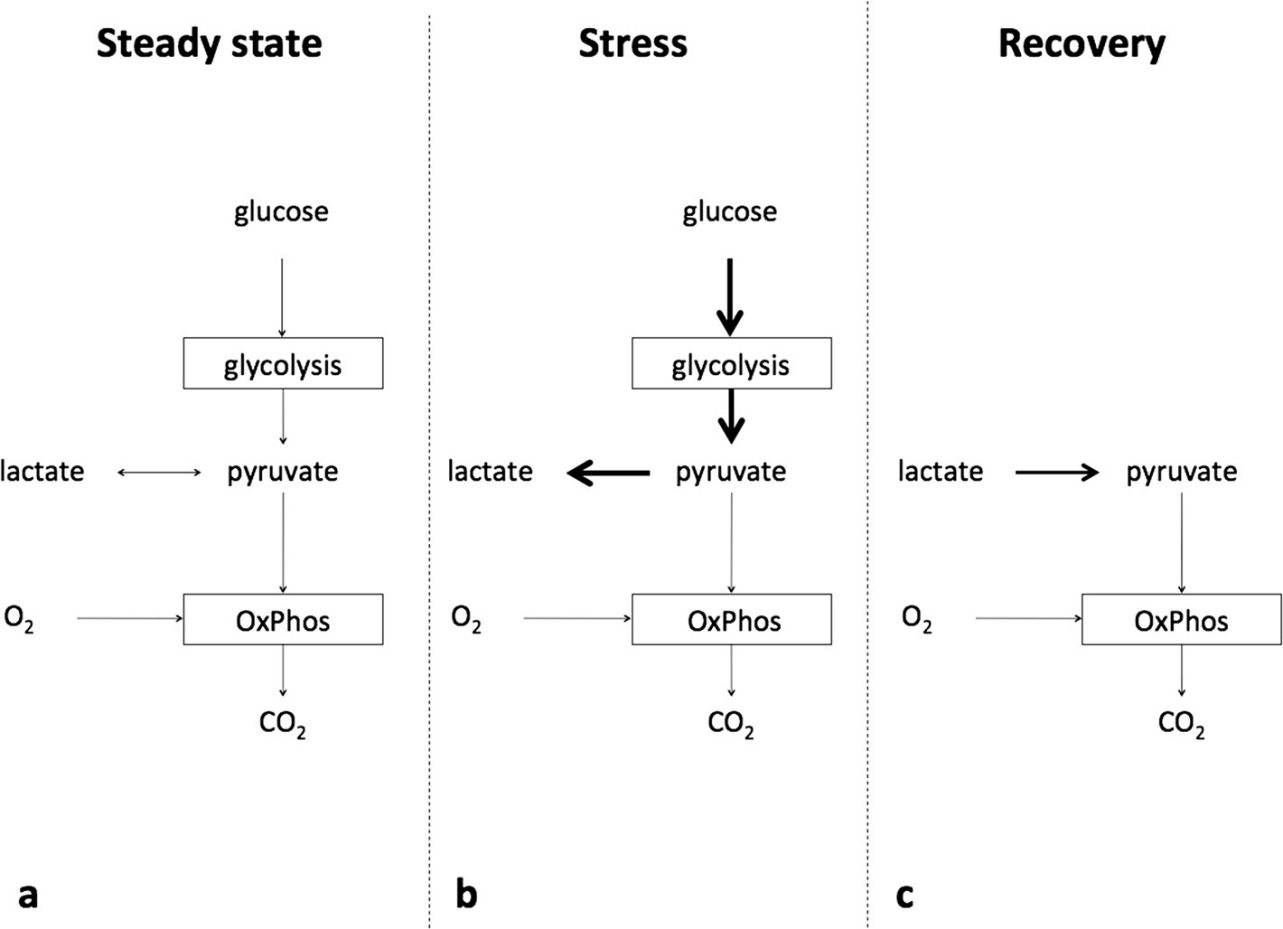
**Lactate at the cellular level.** Usually not oxygen shortage per se, but acute energy requirements is a key determinant of lactate levels. **a** Under stable conditions, glucose is converted to pyruvate, generating 2 ATP, and pyruvate is then subsequently fully oxidized to CO_2_ generating ~36 ATP. **b** Under stress, glycolysis can increase by a factor 100 to 1,000, provided that glucose is present and pyruvate is converted to lactate. Irrespective of optimal mitochondrial function and oxygenation, such a rate of pyruvate production will saturate the mitochondrial tricarboxylic acid cycle and oxidative phosphorylation (OxPhos). **c** During recovery, lactate is converted back to pyruvate and fully oxidized.

**Figure 2:**
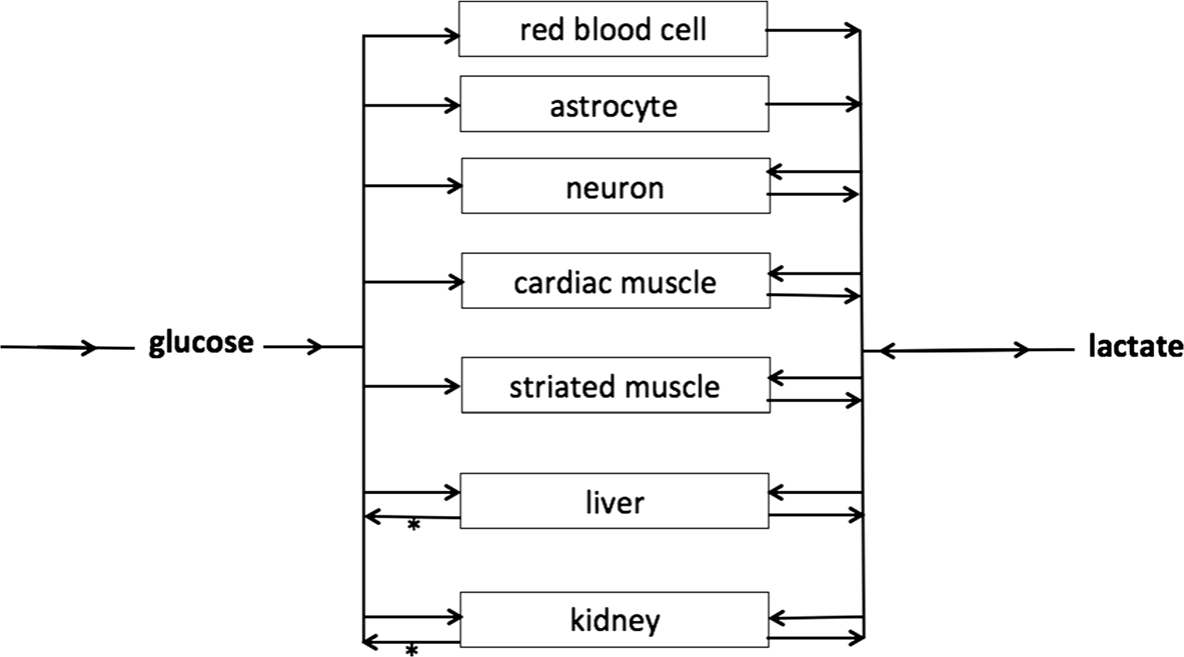
**Lactate at the physiological level.** The flexible use of glucose and lactate as fuels on the cellular level is mirrored at the organism level. All living tissues can consume glucose. From the glucose/lactate point of view, three sorts of tissues/cells exist: 1) cells that must produce lactate because they lack mitochondria, e.g., red blood cells; 2) tissues or cells that either produce or consume lactate depending on circumstances, i.e., all mitochondria-containing cells; 3) tissues that can perform gluconeogenesis and export glucose that is resynthesized from lactate. The liver and the kidneys can only perform gluconeogenesis and export glucose. Only this so-called Cori cycle (denoted by *) carries an energy penalty, whereas the other shuttles do not lead to “waste” of energy.

Lactate is a crucial metabolite in the two main energy (ATP)-producing processes that power life: glycolysis and oxidative phosphorylation (OxPhos). Glycolysis, a process that occurred very early in evolution (approximately 3 billion years ago), converts glucose into two molecules of pyruvate with the concomitant generation of 2 ATP. When atmospheric oxygen levels rose (1 billion years ago), mitochondria developed to generate far more energy from glucose (36 ATP molecules for 1 glucose molecule), although following a much more complicated process (Krebs cycle and OxPhos). Glycolysis and OxPhos steadily metabolize glucose when conditions are stable (Figure 1a). Pyruvate is the molecule that links these two reactions. Because the rate of glycolysis can increase two to three orders of a magnitude faster than OxPhos, glycolysis can briefly provide far more ATP. Excess pyruvate will rapidly accumulate and is diverted to lactate in order for glycolysis to proceed (Figure 1b). During recovery (Figure 1c) lactate is converted into pyruvate. In both directions this is catalyzed by the ubiquitous enzyme lactate dehydrogenase (LDH). Thus, when rapidly large amounts of energy are required, such as under circumstances of cellular stress, lactate serves as a critical buffer that allows glycolysis to accelerate. Also, at the level of the organism (Figure 2), lactate has a similar role as an intermediate fuel that is readily exchanged between various tissues, facilitated by a family of membrane-bound mono-carboxylate transporters (MCT). Over the past two decades, lactate shuttles between astrocytes, neurons, striated muscle, cardiac muscle, as well as the liver and kidneys have been demonstrated [3,4]. The long-known Cori cycle can now be considered one of many lactate shuttles (Figure 2). It should be noted that whereas the Cori cycle involves energy-consuming hepatic or renal gluconeogenesis to convert lactate into glucose, direct interorgan exchange of lactate itself does not carry an energy penalty and even exogenous lactate may serve as a suitable substrate [3].

### Lactate and acidosis

**Figure 3:**
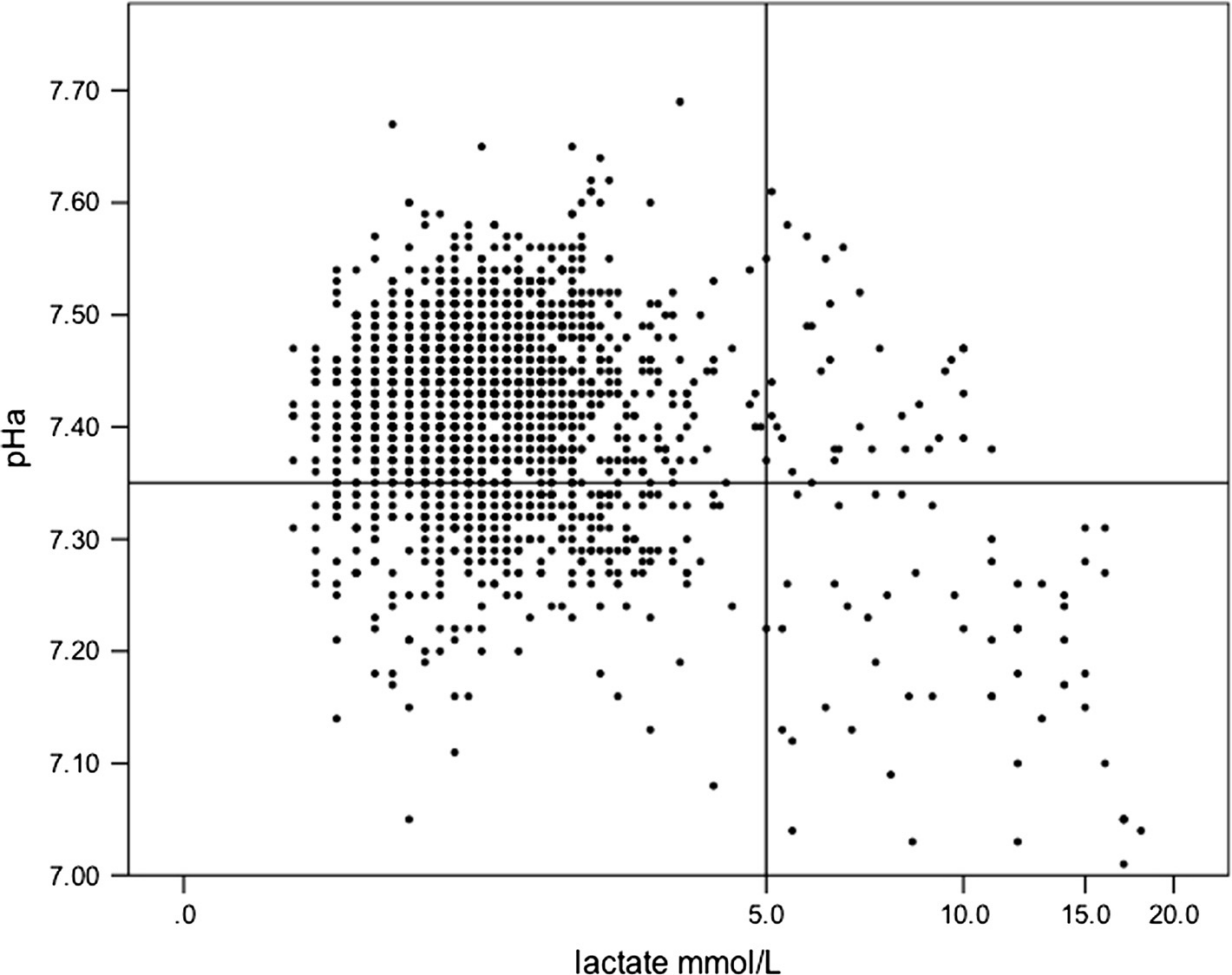
**1,745 combined measurements of arterial pH and arterial lactate in 171 critically ill patients.** Horizontal and vertical lines represent suggested definition of lactic acidosis [8]. For lactate levels ≥ 5.0 mmol/L, a significant linear regression analysis reveals a R^2^ = 0.28 (*p* < 0.001).

The metabolism of glucose during tissue hypoxia results in the production of [lactate]^-^, ATP, and water [5]. The production of H^+^ originates from the hydrolysis of ATP to ADP. In the presence of oxygen and provided that OxPhos can keep up with glycolysis, these H^+^ ions can be used together with lactate in the OxPhos in the mitochondria and acidosis is thus less likely to develop. Stewart challenged the classic Henderson-Hasselbalch approach where the acidosis in his approach is the result of the dissociation of water to maintain acid–base equilibrium by the addition of the strong ion lactate^-^ to the circulation [6]. There is however not a strong relationship between arterial pH and lactate levels. Even at higher lactate levels, only a weak, although significant, correlation exists (Figure 3). When evaluating the significance for patient outcome and the origin of the metabolic acidosis, it is probably more realistic to use the term: lactate-associated metabolic acidosis, a combination that also carries the highest risk of mortality [7].

### Lactate and tissue hypoxia

Many experimental studies have confirmed the relationship between tissue hypoxia and the generation of lactate by reducing the components of systemic oxygen delivery (haemoglobin level, oxygen saturation, and cardiac output) until the extraction of oxygen can no longer maintain oxygen availability to the cells to meet their demands [9,10]. At a critical level of oxygen delivery, oxygen consumption becomes limited by oxygen delivery, and this coincides with a sharp increase in lactate levels. Also, clinical data indicate the relationship between the presence of this supply dependent state of oxygen consumption and increased lactate levels similar to animal studies [11]. In a landmark study, Ronco et al. showed that this phenomenon also was present in patients when oxygen delivery decreased until circulatory arrest during end-of-life care [12]. In addition, Friedman et al. [13] showed that this phenomenon is present in the early resuscitation phase of critical illness, suggesting that resuscitation resolves a state of supply dependent oxygen consumption and thereby the hyperlactataemia. This has been confirmed in an experimental study of cardiac tamponade by Zhang et al. [10], which showed that resolution of the supply-dependent state of oxygen consumption by resolving the tamponade was associated with an increase in oxygen consumption to baseline levels and normalisation of lactate levels.

Because the exchange of oxygen takes place in the microcirculation, alterations in microcirculatory perfusion also can result in limited oxygen availability. Particularly in sepsis, microcirculatory derangement or shunting may lead to insufficient oxygen that is delivered to the cell, thereby increasing lactate levels [10]. This is indirectly illustrated by the observation that improving capillary perfusion has been correlated to a reduction in lactate levels in patients with septic shock, independent of changes in systemic haemodynamic variables [14].

Given the near equilibrium reaction between lactate and pyruvate and its connection with the cellular oxido-reduction state, the lactate-to-pyruvate ratio (L:P) provides additional information as L:P is coupled to the cytoplasmic NADH:NAD+ [15-17]. However, it must be noted that in contrast to lactate, pyruvate is far from trivial to reliably measure in clinical practice and therefore its use is limited in critically ill patients [18].

### Lactate production in aerobic metabolism

**Figure 4:**
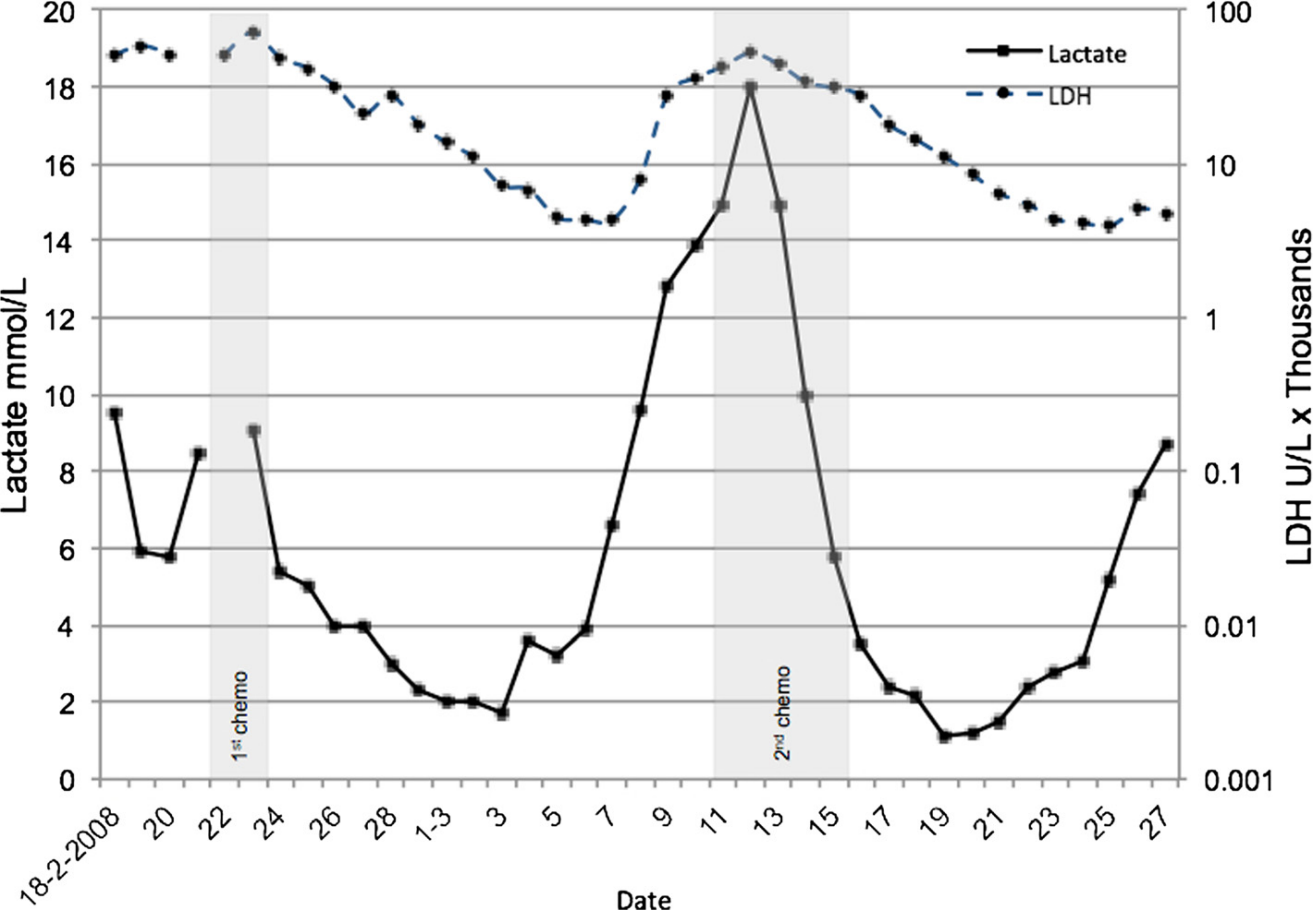
**Lactate levels and LDH levels in a patient with a lymphoma admitted to the ICU because of respiratory failure.** Following diagnosis, treatment with chemotherapy was started. The effect of the first and second chemotherapy on lactate and LDH is shown.

As discussed earlier, aerobic glucose metabolism to lactate may be a preferred way to rapidly produce significant energy amounts. Therefore, stimulating increased aerobic glucose metabolism has been shown to increase lactate levels in the absence of tissue hypoxia. Most notably, the administration of epinephrine has long been shown to result in a dose-dependent increase in lactate levels [19]. Also, stimulation of the phosphofructokinase enzyme by alkalosis (respiratory and metabolic) has been shown to increase lactate levels [20]. Clinically often-used therapeutic interventions also have been shown to increase aerobic lactate production [21]. The aerobic production of lactate as an energy source is related to the very high lactate levels found in patients with lymphoma, a phenomenon referred to as the Warburg effect [22]. When treating the lymphoma, both lactate levels and LDH respond to chemotherapy (Figure 4). Recently, it has been shown that the activity of the Na^+^/K^+^ pump system, which requires significant amounts of ATP for its function, is related to increased lactate levels in both experimental and clinical conditions [23,24], unrelated to the presence of tissue hypoxia. Such enhanced glycolysis can be triggered by cytokine-mediated uptake of glucose [25] or catecholamine-stimulated increased Na-K-pump activity [26,27], supported by both experimental and clinical studies [23,28]. A recent, still unresolved discussion, has focused on the presence of mitochondrial dysfunction in critically ill that could limit pyruvate metabolism (and thus increase lactate levels) in the absence of limited oxygen availability [29,30].

Infusion of Ringer’s lactate does not hamper the accuracy of lactate measurement [31]. Finally, renal replacement therapy eliminates only negligible amounts of lactate [32], but using lactate-containing buffer solutions can induce transient hyperlactataemia [33,34]. Other causes of increased lactate levels (probably) not related to the presence of tissue hypoxia have been described [35-39]. In the case of ethylene glycol intoxication, falsely increased measured lactate levels may result from an adverse reaction to the lactate electrode [40]. Thus, a normal lactate level in the laboratory in contrast to a high level in a point of care device may even be diagnostic in these cases [41].

### Clearance of lactate

Corresponding with its versatile functions, the body is able to clear large lactate loads as shown following the rapid decrease in lactate levels following exercise or return of circulation in cardiac arrest. Likewise, the body is equally adept in clearing very large exogenous loads of lactate during high-volume continuous veno-venous hemofiltration [3,33,42].

Several clinical conditions have been associated with impaired clearance of lactate. First liver dysfunction has been shown to impair lactate clearance [37,43]. Second, in patients following cardiac surgery lactate clearance also may be impaired [44]. Third, in addition to increased glucose metabolism and thus lactate production, sepsis may impair lactate clearance by the inhibition of the rate limiting enzyme pyruvate dehydrogenase [45]. Although this enzyme can be stimulated by dichloroacetate, thus forcing lactate and pyruvate into mitochondrial oxidation, clinical studies have not shown benefit of this increased lactate clearance [46].

### How and where to measure lactate levels

Blood lactate levels can be measured using various devices (central laboratory, point-of-care blood gas analysers, hand-held devices) and generally most devices used at the bedside have acceptable limits of agreement compared to the laboratory devices [47,48]. In addition, the sampling site of the blood (arterial, venous, capillary, etc.) also does not seem to affect the results much [49-51]. However, especially when targeting changes in lactate levels in relatively short intervals, it is not appropriate to use devices and sampling site interchangeably. To prevent an in vitro rise in blood lactate levels, especially when leucocytosis or a high haematocrit (red blood cells do not have mitochondria) are present, a maximum turnaround time of 15 min or storage of the sample on ice is advised [52-54]. Alternatively, tubes containing fluoride, a potent inhibitor of in vitro glycolysis, are widely used.

### Prognosis

Since its first description in humans, increased blood lactate levels have been related to morbidity and mortality. In a recent health technology assessment on the use of lactate levels in critically ill patients, we showed that both in the emergency department and in the ICU blood lactate levels have a place in risk-stratification [55]. Not only one point in time measurements are related to outcome but also the duration and area under the curve of increased lactate levels are related to both morbidity (organ failure) and mortality in different patient groups [56,57]. In the early phase of resuscitation, lactate levels seem to be more closely related to outcome than frequently used haemodynamics, including oxygen delivery and oxygen consumption [58-61]. Moreover, a holistic view incorporating many parameters may be more appropriate in early resuscitation [62].

### Lactate as a goal of therapy

The latter observations stress the importance to define adequate resuscitation goals. Although generally believed to be inadequate, mean arterial pressure is frequently used as an important diagnostic and goal of therapy in patients with haemodynamic instability [63]. Given its strong relationship to the occurrence of inadequate tissue oxygenation and its long-time established relationship with morbidity and mortality, lactate levels could represent a useful goal of initial resuscitation in many clinical conditions. Until recently, the only known single-centre clinical trial advocating such lactate-directed therapy was performed in postcardiac surgery patients [64]. This study showed a reduction in morbidity but was not powered to study the effect on mortality. In addition, translating these findings to a more general and frequently much sicker population is difficult. However, in 2010, two multicentre clinical trials were published on the clinical value of lactate-directed therapy studying a specific group of patients (sepsis) in the Emergency Department [65] and a heterogeneous group of patients with increased lactate levels, not likely to be associated with confounding factors in lactate metabolism, in the ICU [66].

#### Lactate-guided therapy: the United States

In a multicentre, open-label, randomized controlled study, 300 patients were randomized to test the noninferiority between lactate clearance (≥10%) and central venous oxygen saturation (ScvO_2_ ≥70%) as goals of early resuscitation in patients presenting to the ED with severe sepsis or septic shock [65]. The intervention lasted until either all goals were achieved or 6 hours after start of the study. There were no differences in treatments administered during the initial 72 hours of hospitalization. In-hospital mortality in the lactate group was noninferior to the ScvO_2_ group. Although one might conclude that thus both lactate levels and ScvO_2_ are equally effective as a goal of therapy, the study has some limitations that prohibit this. First, venous oxygen saturation might help to differentiate anaerobic from aerobic hyperlactataemia probably resulting in a different treatment [67]. Second, it is questionable whether a 10% reduction in lactate in 6 hours represents an effective resuscitation. The study would implicate that a patient is adequately treated when the initial lactate level of 5.0 mmol/L decreases to 4.5 mmol/L after 6 hours of treatment. Also, a decrease of this magnitude even in the first hour of treatment is not likely to be associated with survival as early resuscitation studies have shown that survivors show an almost 30% decrease in lactate levels in the first hour [68]. Probably the failure to decrease lactate levels at all in response to treatment has more implications for both treatment and prognosis [59,68]. Finally, as only 10% of the patients received either dobutamine or red blood cell transfusion and fluids and vasopressors were guided by CVP and MAP in both groups, the potential difference in protocol actions directly attributable to either lactate or ScvO_2_ was very small. Therefore, it seems unlikely that a change in this resuscitation target could increase mortality by 10%, the noninferiority margin selected for the trial [69].

#### Lactate-guided therapy: the Netherlands

In a multicentre, open-label, randomized, controlled trial, 348 patients were randomly allocated to either lactate-guided treatment (lactate group) or nonlactate-guided treatment (control group) during the first 8 hours of ICU stay [66]. In the lactate group, the treatment goals were a 20% or more decrease in lactate levels per 2 hours and the normalization of ScvO_2_ (>70%). In the control group, lactate levels were not available to the treating physicians during the first 8 hours except for the admission level required for randomization. An important addition to the protocol treatment was the administration of a vasodilator when ScvO_2_ levels where normal but lactate levels did not decrease sufficiently. This is the first study to address this important problem in the resuscitation of critically ill patients, because normalisation of ScvO_2_ is generally regarded as a restoration of the balance between oxygen delivery and oxygen demand that should result in normalization of lactate levels as demonstrated by Zhang et al. [70]. Assuming abnormal microcirculatory perfusion in a state like this that could be improved by the administration of nitroglycerine [71] led to the addition of this intervention to the protocol treatment. We recently showed that nitroglycerine improves abnormal tissue oxygenation in critically ill patients supporting this concept [72]. For a recent, double-blinded, randomized study by Boerma et al., [73] nitroglycerin administration was used in addition to a standard resuscitation protocol in all patients in the protocol group. This study showed no differences in the microcirculation between the two groups and a trend towards an increased mortality in the protocol group. The use of nitroglycerin this study is very different from its use in the study by Jansen et al. [66]. In the latter study, the use of nitroglycerin was guided by a clinical problem (no adequate decrease in lactate levels despite optimal balance between oxygen delivery and oxygen demand), whereas in the study by Boerma et al. nitroglycerin also was used in patients with normal microcirculation, decreasing, or even normal lactate levels, etc. We therefore do not advocate adding nitroglycerin to the standard resuscitation protocols.

Just like in the landmark study on early goal directed therapy [74], protocol patients received significantly more fluids during the intervention period, whereas these patients received less fluid during the subsequent observation period. In addition, significantly more patients were treated with vasodilators (predominantly nitroglycerine) in the protocol group. These differences in treatment were associated with an almost statistically significant (*p* = 0.067), 20% relative reduction in mortality in addition to a strong statistically significant reduction in morbidity (duration of mechanical ventilation and ICU stay, *p* = 0.006).

However, despite the outcome benefit, the course of lactate levels in the two groups was similar. This in fact suggests no causal relationship between the resuscitation therapy and hyperlactataemia. Instead, lactate might be an epiphenomenon of severity of disease. By acting as a warning signal, clinicians might have interpreted hyperlactataemia as a warning that their patients did not clinically improve or even deteriorate in the presence of stable haemodynamic parameters. This could have triggered additional diagnostic and therapeutic interventions.

Summarizing, these two recent studies show that lactate-directed resuscitation therapy has clinical benefit for critically ill patients, although the exact mechanism behind it remains uncertain. Lactate measurement probably should be accompanied by venous saturations monitoring to guide decision-making and therapy.

## Conclusions

To understand the importance of an increased lactate level, it is important not only to consider anaerobic production but also aerobic mechanisms and changes in lactate clearance. Despite this complex evaluation, increased lactate levels usually reflect increased morbidity and high mortality. In addition, two recent multicentre trials suggest that the use of lactate levels in goal-directed therapy may improve clinical outcome. These findings confirm that lactate monitoring is a valuable parameter in the early resuscitation of critically ill patients.

## Competing interests

The authors declare that they have no competing interests.

## Authors’ contributions

JB, MWNN and TCJ all contributed to the paper and wrote equal sections of the manuscript. JB did the final editing of the manuscript. All authors read and approved the final manuscript.
